# Complicated Gitelman syndrome and autoimmune thyroid disease: a case report with a new homozygous mutation in the SLC12A3 gene and literature review

**DOI:** 10.1186/s12902-018-0298-3

**Published:** 2018-11-08

**Authors:** Haiyang Zhou, Xinhuan Liang, Yingfen Qing, Bihui Meng, Jia Zhou, Song Huang, Shurong Lu, Zhenxing Huang, Haiyan Yang, Yan Ma, Zuojie Luo

**Affiliations:** The Department of Endocrinology, The First Affiliated Hospital of Guangxi Medical University, Nanning, 530021 China

**Keywords:** Gitelman syndrome, Graves’ disease, Hypokalemia

## Abstract

**Background:**

Gitelman syndrome (GS) is an inherited autosomal recessive renal tubular disorder characterized by low levels of potassium and magnesium in the blood, decreased excretion of calcium in the urine, and elevated blood pH. GS is caused by an inactivating mutation in the SLC12A3 gene, which is located on the long arm of chromosome 16 (16q13) and encodes a thiazide-sensitive sodium chloride cotransporter (NCCT).

**Case presentation:**

A 45-year-old man with Graves’ disease complicated by paroxysmal limb paralysis had a diagnosis of thyrotoxic periodic paralysis for 12 years. However, his serum potassium level remained low despite sufficiently large doses of potassium supplementation. Finally, gene analysis revealed a homozygous mutation in the SLC12A3 gene. After his thyroid function gradually returned to normal, his serum potassium level remained low, but his paroxysmal limb paralysis resolved.

**Conclusions:**

GS combined with hyperthyroidism can manifest as frequent episodes of periodic paralysis; to date, this comorbidity has been reported only in eastern Asian populations. This case prompted us to more seriously consider the possibility of GS associated with thyroid dysfunction.

## Background

Gitelman syndrome (GS) is an inherited autosomal recessive renal tubular disorder characterized by low levels of potassium and magnesium in the blood, decreased excretion of calcium in the urine, and elevated blood pH. GS is caused by an inactivating mutation in the SLC12A3 gene, which is located on the long arm of chromosome 16 (16q13) and encodes a thiazide-sensitive sodium chloride cotransporter (NCCT). Graves’ disease (GD) is a common cause of hyperthyroidism. In our department, we diagnosed a patient with GD and GS.

## Case presentation

The patient was a 45-year-old male with a 12-year history of paroxysmal weakness of the limbs. He was diagnosed with hypokalemic periodic paralysis in 2005 and hyperthyroidism in 2008. He had taken antithyroid drugs on an irregular basis since 2008 but had not undergone proper biochemical examination. Whenever he felt that his weakness was becoming severe, he would self-prescribe potassium chloride. In June 2017, the extent of his lower limb weakness increased such that he could no longer walk. He took potassium chloride without improvement. Subsequently, he was admitted to another hospital. His temperature was 36.7 °C, and his pulse was 96 beats/min. The muscle strength in his lower limbs was grade II [1], and that in his upper limbs was grade III. His limb muscle tone was normal. His electrolyte and blood marker levels were as follows: K^+^, 1.4 mmol/l; Na^+^, 138 mmol/l, Cl^−^, 97 mmol/l; Ca^2+^, 2.61 mmol/l; free triiodothyronine (FT3) 6.96 pmol/l (1.86–6.44); free thyroxine (FT4) 38.96 mIU/l (11.45–22.14); thyroid-stimulating hormone (TSH) < 0.01 mIU/l (0.4–4.5); thyroglobulin antibody (TgAb) 16.61 IU/ml (0–150); and thyrotropin receptor antibody (TRAb) 22.36 mIU/l (0–5). Thyroid ultrasound demonstrated diffuse thyromegaly with a rich blood supply. The patient was diagnosed with GD and hypokalemic periodic paralysis and was treated with propylthiouracil (PTU) and potassium chloride. However, 2 days later, despite improvement of his weakness, his temperature increased to 41 °C, and he experienced cough and expectoration. Computed tomography (CT) imaging of his lungs revealed pneumonia. He was subsequently treated with cefazolin and transferred to our hospital 2 days later.

When the patient was admitted to our department, his limb weakness had significantly improved. He had a temperature of 38.8 °C, a pulse of 96 beats/min, a breathing rate of 20 respirations/min, a blood pressure of 106/68 mmHg, and grade II thyroid enlargement. Vascular murmur was audible in the thyroid. The muscle strength in his limbs was grade V, and his limb muscle tone was normal. The patient’s biochemical parameters were as follows (the reference values are different from those used in the previous department): [blood count] leukocytes 13.10 × 10^9^/l, neutrophils 11.99 × 10^9^/l and hemoglobin 13.3 g/dl; [serum electrolytes] K^+^ 2.110 mmol/l, Na^+^ 131.6 mmol/l, Cl^−^ 91.1 mmol/l, Ca^2+^ 1.850 mmol/l, and Mg^2+^ 0.540 mmol/l; [thyroid function and thyroid antibodies] triiodothyronine (T3) 1.40 mmol/l (1.34–2.75), thyroxine (T4) > 300 nmol/l (78.38–157.40), FT3 5.32 pmol/l (3.60–6.00), FT4 51.23 pmol/l (7.86–14.41), TSH 0.01 mIU/l (0.34–5.65), thyroid peroxidase antibody (TPOAb) 36.33 IU/ml (0–30), TRAb 9.011 IU/ml (0–30), TgAb 6.04% (< 30%), and thyroid microsomal antibody (TMAB) 6.48% (< 20%); and creatine kinase (CK) 1398 U/l (38–174) and CK-MB 29 U/l (0.0–25.0). The patient’s liver and kidney functions were normal. We treated him with cefazolin, propranolol, PTU and potassium chloride. The patient’s vital signs and strength normalized after 3 days, and his leukocyte count had decreased to 5.97 × 10^9^/l, his neutrophils had decreased to 3.59 × 10^9^/l, and his CK had decreased to 40 U/l. However, his serum potassium level remained low despite 24 g/d of potassium supplementation. Additionally, the patient had hypomagnesemia and metabolic alkalosis (the results are shown in Tables 1 and 2). Further testing showed that his renin activity (supine) was 5.17 ng/ml/h (reference value 0.15–2.33), his aldosterone level was 436.10 pg/ml (10–160), his random urinary calcium/creatinine ratio was 0.23, his osteocalcin level was 1.06 ng/ml (6.00–48.00), his parathyroid hormone level was 11.22 pg/ml (6.0–80.0) and his calcitonin level was 4.87 pg/ml (0.00–18.00).

**Table 1:** Serum and urine electrolyte levels on admission

	24th July		5th August	
	Urine electrolytes	Serum electrolytes	Urine electrolytes	Serum electrolytes
K (mmol/l)	214.05	2.850	245.71	3.550
Ca (mmol/l)	4.80	2.303	2.91	2.309
Mg (mmol/l)	6.40	0.720	3.35	0.54
Urine volume (ml)	3200	–	3100	–

**Table 2:** Blood gas analysis on admission

	22nd Jul	24th Jul	4th Aug
pH	7.602	7.499	7.447
PCO_2_	26.1	31.9	37.7
PO_2_	78.3	91.3	93.0
HCO_3_^−^	25.2	24.3	25.4
BE	5.0	1.8	1.6
K^+^	1.92	2.72	2.67
Ca^2+^	1.03	0.97	1.03
Cl^−^	93	102	100

**Fig. 1:**
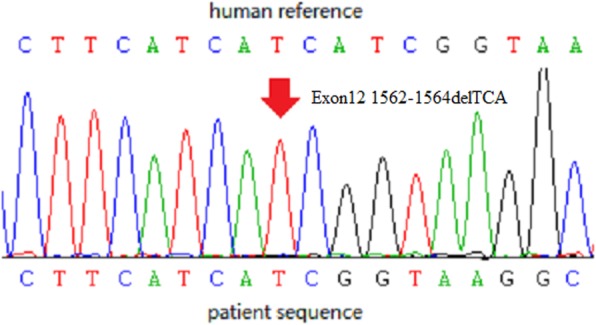
NGS sequencing of the SLC12A3 gene fragment encompassing homozygous mutation c.1562_1564delTCA in patient

**Fig. 2:**
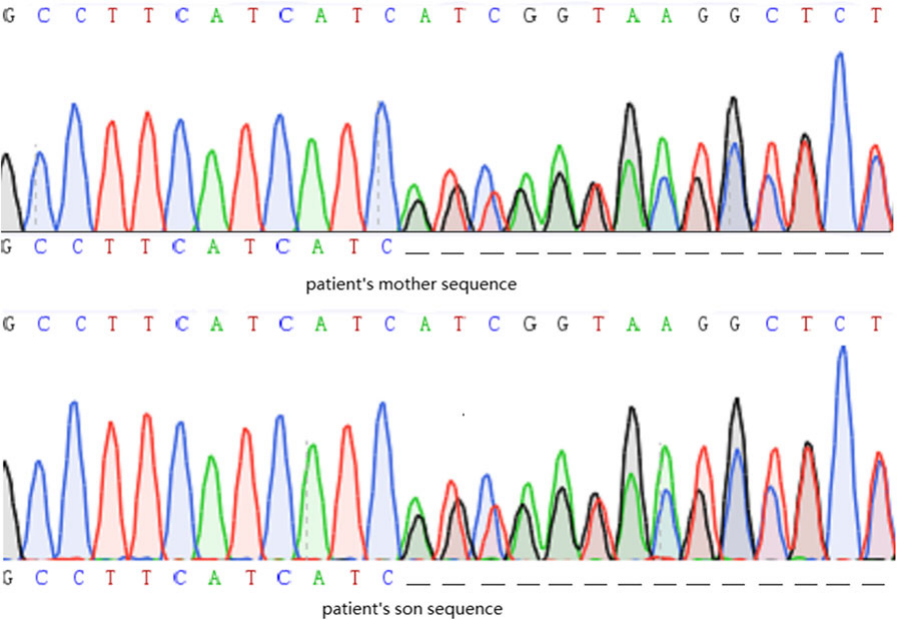
Sanger sequencing of the SLC12A3 gene fragment encompassing heterozygous mutation c.1562_1564delTCA in patient mother(above) and

Based on these results, we suspected that the patient did not have thyrotoxic periodic paralysis (TPP) but rather GS. Therefore, we sent a blood sample to Beijing Huada Company for sequencing. The Next Generation Sequencing (NGS) was used. The sequencing protocol was based on the Roche Nimblegen SeqCap EZ Choice XL Library for exon trapping. A total of 25 genes (Table 3) known to be associated with hypokalemia were targeted and the total size of target regions was 11.8 M. Libraries were prepared with the Kapa Hyper Prep kit and sequencing was carried out by Illumina NextSeq500 System. The sequencing data were compared to the human genome by BWA (0.7.12-r1039) software (http://bio-bwa.sourceforge.net/), and ANNOVAR (Date: 2015-06-17) was used to annotate the mutation sites based on dbSNP, Clinvar, ExAC, and 1000 genomes, among others. We found a homozygous mutation in the SLC12A3 gene (Exon12 1562-1564delTCA) with an amino acid change of 522delIle, which was first reported as a compound heterozygous mutation.by Vargas-Poussou [2]. The mutation was confirmed by sanger sequencing. No other phenotypes were found, including those for Bartter syndrome, hypokalemic periodic paralysis,Liddle syndrome, hyperaldosteronism, and apparent mineralocorticoid excess. The diagnosis was changed to GD with GS. Moreover, we obtained blood samples from the patient’s mother and son (his father had passed away) who did not have hypokalemia and hyperthyroidism. Both of them were proved as heterozygous mutation carriers by sanger sequencing. The sequencing chromatograms are shown in Figs. 1 and 2. The patient had three brothers and one sister, but we were unable to obtain blood samples from them.

**Table 3:** The list of genes in the panel

SLC12A3	SCN4A	KCNJ2
SLC12A1	HSD11B2	ATP6V0A4
KCNJ1	KCNJ5	KCNJ10
CLCNKB	SCCN1B-exon13	SLC34A1
BSND	SCCN1G-exon13	EHHADH
CLCNKA	CYP11B1	HNF4A
CASR	CTP17A1	SLC4A1
CACNA1S	NR3C1	
KCNE3	CACNA1D

In addition to antithyroid drugs (methimazole 30 mg/d), we gave the patient potassium chloride (3 g/d), potassium citrate (6 g/d), and magnesium potassium aspartate (1.788 g/d). At the follow-up visit, we found that the patient often forgot to take his medicine. The results for thyroid function and electrolyte levels before and after treatment are listed in Table 4, which indicated that the patient’s thyroid function had improved. Hypothyroidism occurred during the course of treatment, but the patient’s thyroid function returned to normal after we reduced the dose of methimazole. The patient refused the recommendation to undergo I^131^ therapy. His serum potassium level remained low despite a sufficiently large daily dose of potassium, but no paroxysmal paralysis occurred after discharge.

**Table 4:** Most recent re-examination results

	SerumK^+^ (mmol)	Serum Mg^2+^ (mmol)	T3 (nmol/l)	T4 (nmol/l)	FT3 (pmol/l)	FT4 (pmol/l)	TSH (mIU/l)
2017.22nd Jul (admission)	2.110↓	0.540↓	1.40	> 300↑	5.32	51.23↑	0.01↓
5th.Aug	3.550	0.520↓	–	–	–	–	–
18th Aug (discharge)	2.580↓	0.540↓	2.75↑	189.53↑	8.88↑	20.77↑	0.01↓
19th Sep	2.810↓	0.700↓	2.63↑	114.75↑	6.69↑	15.81↑	0.01↓
27th Oct	2.530↓	0.700↓	–	–	4.33	3.85	1.39↓
12th.Dec	2.360↓	0.700↓	–	–	2.64↓	1.87↓	38.87↑
2018.8th.Feb	2.440↓	0.680↓	–	–	4.00	8.46	5.23↑
28th.Mar	–	–	–	–	4.45	9.59	5.43↑

## Discussion and conclusions

More than 400 SLC12A3 variations have been identified to date. The prevalence of GS among Japanese populations is 10.3/10000 [3, 4]. The incidence rates of GD and Hashimoto’s thyroiditis (HT) among the Chinese population are 120/100000/year and 100/100000/year, respectively [5]. The prevalence rates of both autoimmune thyroid disease (AITD) and GS among East Asian populations are higher than those among European populations [6]. Through a Chinese and English literature review, we identified 17 cases of AITD complicated with GS from nine papers (18 cases including ours) [7–17]. The cases included seven males (aged 20~ 45 years) and 11 females (aged 14~ 50 years). Among the patients, 13 had GD, 3 had HT, and two had antibody-positive AITD. All patients with GD and HT developed hypokalemic periodic paralysis. Twelve patients underwent genetic analysis, and all mutations were located in the SLC12A3 gene; four patients were homozygotes, one was a heterozygote, and five were complex heterozygotes. One patient did not have any detectable mutation, and the mutation type for one patient was not mentioned. The details of these cases are listed in Tables 5 and 6. Except for our case, the mutations in all cases were single base substitutions; two of the mutations were T60 M [18], which is a common variation among Chinese individuals. Six patients underwent renal biopsy, and all of the patients had juxtaglomerular complex (JGC) hyperplasia. Patient 6 had clinical and pathological features of GS but a wild-type SLC12A3 gene; therefore, the patient may have had acquired GS caused by autoimmune disease [19, 20]. Interestingly, we noticed that all the reported cases were from eastern Asia, possibly because of the high prevalence of GS and AITD in East Asian populations.

**Table 5:** Pathology and gene information for the reported cases to date

No	Sex	Age	Thyroid disease	Diagnosis method	Pathology	Mutation type	Variation site	Change in nucleotide base	Change in amino acid
1	Male	45	GD	Gene analysis	No mention	Homozygote	Exon 12	1562-1564delTCA	522delIle
2 [7]	Male	39	GD	Gene analysis	No mention	Compound heterozygote	Exon 15	1841C-T	Ser614Phe
							Exon 26	2968G-A	Arg990Lys
3 [7]	Female	41	Antibody-positive	Gene analysis	No mention	Compound heterozygote	Intron 7	964 + 2 T-C	
							Exon 1	179C-T	Thr60Met
4 [8]	Female	40	Antibody-positive	Gene analysis	No mention	Compound heterozygote	Exon 22	2552 T-A	Leu849His
							Exon 22	2561G-A	Arg852His
5 [8]	Female	28	GD	Gene analysis	No mention	Homozygote	Exon 22	2552 T-A	Leu849His
6 [9]	Male	20	HT	Pathology and Gene analysis	JGC hyperplasia	Wild-type	–	–	–
7 [9]	Female	46	GD	Gene analysis	No mention	Heterozygote	Exon 1	185C-T	Thr60Met
8 [9]	Male	21	GD	Gene analysis	No mention	Homozygote	Exon 23	2744G-A	Arg913Gln
9 [10]	Female	18	GD	Gene analysis	No mention	Compound heterozygote	Exon 12	1015A-C	Thr339Pro
							Exon 22	2573 T-A	Leu858His
10 [10]	Female	50	GD	Gene analysis	No mention	Compound heterozygote	Exon 4	539C-A	Thr180Lys
							Exon 8	1045C-T	Pro349Ser
11 [10]	Female	56	GD	Gene analysis	No mention	Homozygote	Exon 14	1706C-T	Ala569Val
12 [11]	Female	14	GD	Gene analysis	No mention	No mention	Exon 6	791G-C	Gly264Ala
13 [12]	Female	20	GD	Pathology	JGA hyperplasia	–	–	–	–
14 [13, 14]	Male	21	GD	Pathology	JGA hyperplasia		–	–	–
15 [14]	Male	18	GD	Pathology	JGA hyperplasia			–	–
16 [15]	Female	39	HT	Biochemical analysis	–			–	–
17 [16]	Male	22	GD	Pathology	JGC hyperplasia				
18 [17]	Female	29	HT	Pathology	JGC hyperplasia

**Table 6:** Chemical indicators of the reported cases to date

No	Serum potassium (mmol/l)	Serum magnesium (mmol/l)	Urinary calcium/creatinine	pH	HCO_3_-	FT3	FT4	T3	T4	TSH (mIU/l)	TPOAb	TRAb
1	1.4	0.54	0.23	7.602	25.2	↑	↑	↑	↑	0.1	↑	↑
2	1.9	0.52	0.08			↑	↑	↑	↑	0.01	–	↑
3	2.6	0.4	0.01			normal	normal			2.22	↑	
4	3.3	1.8	0.029	7.458	29.3	normal	normal			2.02	↑	
5	1.7	1.5	0.02	7.506	35.4	↑	↑			0.01		↑
6	2.67	0.56	0.05		29	↓	↓			75.4	↑	
7	2.3	0.43	0		33	↑	↑			0.01	↑	↑
8	2.64	0.36	0.07		35	↑	↑			<0.005	↑	↑
9	3.2	0.86	<0.003			–	↑			0.01		normal
10	3	0.66	<0.003			–	↑			<0.005		↑
11	2.8	0.49	0.03			–	↑			0.007		↑
12	2.2	0.53				↑	↑			0.01	↑	↑
13	2.46	0.73		7.47								
14	1.87			7.45	27	↑	normal	↑	normal	–		
15	2.02	0.5		7.55	28	↑	↑	↑	↑	–		
16	2.44	0.45		7.488	27.3			normal		4.28		
17	1.8	0.49–0.53		7.46	26.6	↑	↑			0.01	normal	
18	2.65	0.55		7.45	32.6	normal	normal			66.78	↑	

Nevertheless, we still lack sufficient data to demonstrate whether AITD is more likely to occur in patients with GS. Patients with GS may undergo more extensive testing, including thyroid functional analysis, compared to healthy individuals, which may facilitate the identification of additional abnormalities. Although sufficient data are available regarding the induction of hypokalemia and hypomagnesemia by hyperthyroidism, research on the long-term effects of hypokalemia and hypomagnesemia on the thyroid is lacking. Iodine and magnesium metabolism have been found to be closely linked [21]. One study showed that long-term high dietary magnesium can lead to abnormal thyroid function. Another study suggested that hypomagnesemia may lead to rapid relapse of GD [22]. In contrast, increasing magnesium supplementation has also been shown to promote normalization of thyroid morphology and function [23].

Autoimmune thyroid diseases (AITDs) are complex genetic diseases. The genes contributing to AITD can be divided into two categories: immunomodulatory genes, including the human leukocyte antigen (HLA), cytotoxic T lymphocyte-related antigen 4 (CTLA-4), protein tyrosine phosphatase, nonreceptor type 22 (PTPN22), CD40, CD25, and Fc receptor-like 3 (FCRL3) genes, and thyroid-specific genes, including the thyroid-stimulating hormone receptor (TSHR) gene and the thyroglobulin (Tg) gene. However, no studies have indicated that a correlation exists between these genes and the SLC12A3 gene.

In conclusion, GS combined with hyperthyroidism (or other AITDs) can cause hypokalemic periodic paralysis. Our patient was misdiagnosed with hypokalemic TPP for a long time, indicating that the possibility of GS should be considered in clinical cases with hyperthyroidism and persistent hypokalemia.

## Abbreviations

AITD: autoimmune thyroid disease
CT: computed tomography
FT3: free triiodothyronine
GD: Graves’ disease
GS: Gitelman syndrome
HT: Hashimoto’s thyroiditis
JGC: juxtaglomerular complex
PTU: propylthiouracil
TgAb: thyroglobulin antibody
TMAB: thyroid microsomal antibody
TPOAb: thyroid peroxidase antibody
TRAb: thyrotropin receptor antibody
TSH: thyroid-stimulating hormone
WBC: white blood cell
